# Vision-Based Person-Following Algorithm for Assistive Elderly-Care Quadruped Robots

**DOI:** 10.3390/s26103263

**Published:** 2026-05-21

**Authors:** Vishnudev Kurumbaparambil, Subashkumar Rajanayagam, Stefan Twieg

**Affiliations:** Department of Electrical, Mechanical and Industrial Engineering, Hochschule Anhalt, Bernburger Str. 55, 06366 Köthen, Germany; subashkumar.rajanayagam@hs-anhalt.de (S.R.);

**Keywords:** quadruped robot, computer vision, person-following, follow me, Robot Operating System, Unitree Go1, ZED camera

## Abstract

The demographic shift towards an aging population necessitates innovative solutions for care and mobility support. While commercial quadruped robots like the Unitree Go1 offer dynamic stability, their native following modes often lack the safety margins and predictability required, and they do not consistently follow the user, at times deviating and navigating independently. This paper presents a robust, vision-based, person-following algorithm designed to address these limitations. Utilizing a ZED 2 stereo camera and Robot Operating System (ROS), the system employs a finite state machine to ensure deterministic target tracking. A velocity control strategy partitions the robot’s motion into distinct stability, proportional, and braking zones based on depth data to ensure fluid interaction. The framework was validated on a Unitree Go1 quadruped platform in an outdoor environment involving 90-degree turns to evaluate tracking robustness. By operating in a headless mode, the system achieved a mean processing latency of 66.5±4.3 ms. Experimental results demonstrated consistent operational stability, 0.0% intrusion into the intimate safety zone, and effective velocity synchronization between 0.47 and 0.54 m/s. While this study establishes a robust technical baseline using healthy subjects, it serves as a preliminary development platform; further iterative testing with elderly users in clinical settings is required to move toward deployment. Beyond the evaluated trials, the framework maintained reliable functional performance across various care facility workshops, successfully following the target in all deployment scenarios. These findings establish a stable technical foundation for the future development of robotic walking partners.

## 1. Introduction

### 1.1. Motivation

The global increase in the elderly population presents a significant challenge to healthcare systems, particularly in the domain of geriatric care and physical rehabilitation [1,2]. Regular physical activity, such as walking, is crucial for maintaining the physical and mental health of elderly people and is strongly associated with preserved independence and reduced risk of chronic disease [3,4]. However, in residential care facilities and nursing homes, staff shortages often limit the individual supervision available to residents during daily exercise sessions [5,6]. Socially Assistive Robots (SARs) have been proposed to bridge this gap by offering companionship and basic monitoring [7,8,9,10,11]. Beyond social interaction, there is increasing interest in robots that act as “walking partners” to encourage movement and enhance safety. Recent surveys indicate that although the public views quadrupedal robots positively for dementia care, specifically for monitoring and companionship, acceptance is heavily dependent on demonstrated safety and effectiveness [12]. The EduXBot project (Educational Exploration Robot Application Platform) [13] aims to develop such a platform to support the physical and cognitive well-being of elderly individuals. Within this context, the robot must be able to safely follow or accompany the person in environments, such as corridors or parks. Quadruped robots, such as the Unitree Go1, offer distinct advantages over wheeled platforms in these settings due to their ability to traverse irregular terrain, curbs, and ramps.

### 1.2. Problem Statement and Objectives

**Figure 1:**
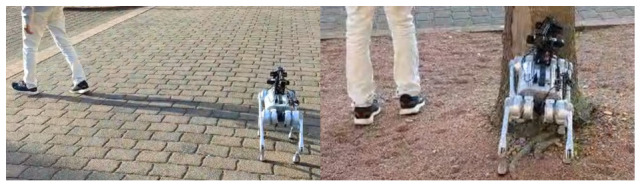
Snapshots from preliminary testing of the Unitree Go1 built-in follow mode.

Commercial quadruped platforms like Unitree Go1 are suitable for research due to programmability, dynamic stability, and support for Robot Operating Systems (ROSs) [14]. It also offers built-in “follow me” functions; however, these are primarily designed for sports or logistics and lack the safety margins and following reliability required for geriatric care. To validate the platform’s suitability for elderly assistance, we conducted preliminary tests using the native “Intelligent Side-Following System” [15]. As illustrated in Figure 1, the native system demonstrated significant instability. The robot failed to settle into a “wait” state when the user stopped. Despite having an obstacle avoidance system built into the Go1, the robot failed to avoid obstacles during the following mode. Furthermore, the mode selection process was unclear, making it difficult to determine if a failure was due to user error or system malfunction. The system also struggled to maintain a consistent distance, occasionally leaving the robot stranded.

**Figure 2:**
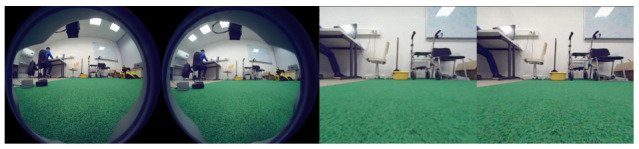
Comparison of the Go1’s internal fisheye camera output (**left**) versus the rectified view (**right**).

Developing a vision-based following system requires reliable sensing and sufficient processing power. The Unitree Go1 contains five sets of built-in fisheye cameras distributed across three internal Jetson computing boards [16]. Due to storage and processing limitations, a specific board is preferred for complex operations. Consequently, accessing images from the remaining cameras requires data transfer between boards via the Unitree Camera SDK, utilizing UDP or ROS topics. Preliminary testing confirmed that this architecture introduces significant latency, rendering the internal cameras too slow for real-time person following. Furthermore, rectifying the fisheye images results in a severe loss of peripheral visual data, as seen in Figure 2. This reduced field of view is insufficient for maintaining a lock on a human target during sharp turns.

Consequently, the native proprietary “black-box” system is inadequate for the safety-critical requirements of geriatric assistance. To address these architectural and performance barriers, this paper proposes a specialized framework that shifts the focus from high-dynamic agility toward deterministic, safe interaction.

### 1.3. Scientific Contributions

The primary contribution of this work is a sensor-driven, depth-based safety and tracking framework that prioritizes deterministic behavior over the high-agility movement typically found in commercial “follow-me” systems. Unlike proprietary “black-box” firmware, this study introduces:

- **Deterministic Tracking and Robust Re-acquisition:** We implement a Finite State Machine (FSM) that ensures predictable transitions between tracking, searching, and waiting states. This architecture provides a robust recovery logic during target loss by combining directional memory with a “Reliability Check” protocol. This protocol utilizes both a Depth Consistency Constraint ($\Delta_{depth}$) and a Geometric Entry Constraint to distinguish the intended target from bystanders, ensuring tracking integrity during the recovery phase.
- **Depth-Based Velocity Zoning:** Rather than relying on standard proportional control, the algorithm partitions motion into stability, proportional, and critical braking zones based on real-time depth data. This design prioritizes motion smoothness for following at a natural human walking speed and preventing high-frequency oscillations.
- **Operational Reliability and Empirical Validation:** The framework was validated through systematic field trials on a Unitree Go1 platform, specifically evaluating tracking robustness during 90° turns and bystander interference to verify a 0.0% intrusion rate into human proxemic zones. Beyond the reported experimental logs, the system demonstrated consistent functional reliability across multiple pilot tests and care facility workshops, successfully maintaining target synchronization in all deployment scenarios. This establishes a stable technical baseline for future iterative development with elderly users.

## 2. Literature Review

### 2.1. Socially Assistive Robots for Mobility

Socially Assistive Robots (SARs) combine assistance with social interaction to provide coaching or cognitive stimulation [7,8,9,10,11]. In the context of mobility support, reviews indicate that older users prioritize robots that move smoothly, behave in a predictable manner, and maintain a comfortable distance [17]. Currently, most mobility robots function as smart walkers or wheelchair companions designed for flat indoor surfaces. Quadruped-based assistants are rare in this field. However, unlike wheeled platforms, legged robots offer the potential to operate effectively in mixed indoor and outdoor terrain.

### 2.2. Person-Following Technologies

While quadrupeds offer superior terrain mobility [18], leveraging this capability requires robust perception systems to maintain contact with the user. Person-following is a foundational capability in service robotics [19,20]. Early systems used 2D laser scanners to detect leg patterns [21,22]. While computationally efficient, these methods are sensitive to crowded or cluttered spaces [23,24]. For robust perception in dynamic environments, assistive robots can employ integrated systems that combine deep learning-based object detection with depth sensing. For instance, implementations utilizing YOLO for real-time identification [25,26,27] and RGB-D cameras like the ZED 2 to obtain precise distance measurements have been successfully deployed on mobile robotic platforms [28,29,30]. However, for the specific demands of elderly care, recent surveys emphasize that while many prototypes exist, few address the robustness required for elderly interaction, where user motion is slow and non-linear and environmental lighting varies significantly.

Generally, tracking relies on either an active method, which requires the user to wear sensors (e.g., UWB or RFID tags) [31,32], or a passive method, such as computing position from camera data. Although active methods provide high precision, they require seniors to wear tags, which they may forget or refuse to wear. This makes passive perception more suitable for seamless interaction.

### 2.3. Vision-Based Tracking on Quadrupeds

Low-cost quadruped robots [33,34], such as the Unitree Go1, are increasingly popular in research. They offer high agility, dynamic stability, and extensive programmability. However, legged locomotion introduces specific challenges for visual perception that are less noticeable in wheeled robots [35,36,37]. The significant body pitch and roll during gait cycles can introduce noise into visual tracking algorithms. Furthermore, commercial “follow” modes often rely on closed-source systems that combine cameras with wireless tags. These proprietary systems typically do not allow for the fine-tuning of safety margins or control dynamics, which is critical for ensuring user comfort in geriatric care applications [38,39,40]. Because of the lack of adaptability and transparency in commercial black-box systems, custom open-source software is necessary to prioritize user safety.

## 3. Methodology

### 3.1. System Architecture

**Figure 3:**
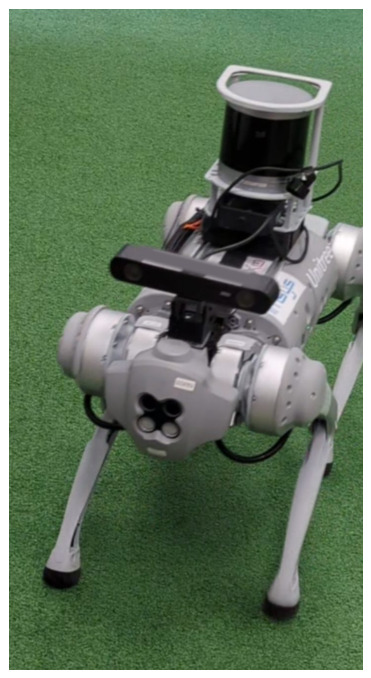
The Unitree Go1 platform equipped with the ZED 2 stereo camera.

To overcome the proprietary hardware limitations identified in Section 1, a custom architecture was designed. The internal fisheye cameras were bypassed in favor of an external ZED 2 stereo camera from Stereolabs. The camera was mounted on the Go in such a way that the human torso remains within the vertical field of view (FoV) at the target following distance (Figure 3).

A key limitation of the onboard fisheye cameras is the reduction in effective field of view after rectification. Based on the camera calibration parameters [16], the original frame resolution of 1856 × 800 pixels is reduced to 928 × 800 pixels after rectification, corresponding to an approximate 50% loss in horizontal visual coverage. This significantly limits peripheral visibility, increasing the likelihood of target loss during lateral motion or sharp turns. In addition, the effective depth-sensing range of the onboard camera system is limited to approximately 0.1–0.85 m [16]. This is insufficient for the current use case of following a human at a distance, as stable perception beyond this range is required. In contrast, the ZED 2 provides a native rectified stereo image with a horizontal FoV of approximately 110° and reliable depth estimation up to 20 m [41], enabling consistent target perception without additional geometric correction.

Note that the LIDAR sensor shown is part of the platform’s SLAM expansion hardware and is not utilized in the vision-based following algorithm described in this study. The ZED 2 provides up to 2K video output and depth maps of up to 20 m. The robot operates on ROS Melodic, while the high-level control software executes on an external laptop running ROS Noetic to offload processing. Communication between the robot and the external laptop is established via the robot’s hosted Wi-Fi hotspot.

**Figure 4:**
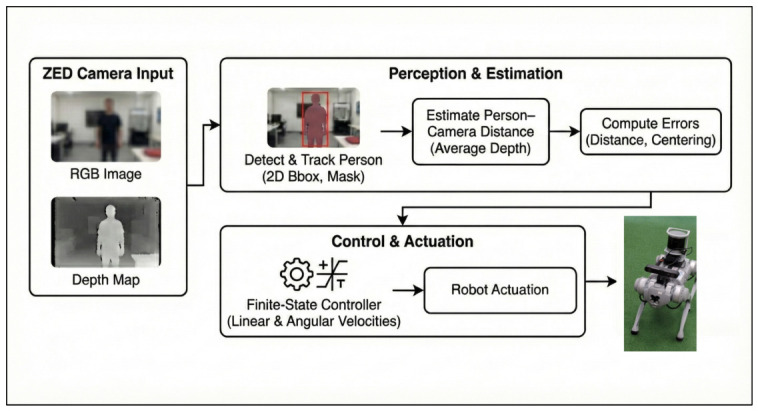
A high-level block diagram of the proposed system architecture.

The control software is implemented in Python 3.8, utilizing the ZED SDK [42] to generate velocity commands communicated via standard ROS topics. The complete system architecture is shown in Figure 4.

### 3.2. State Machine Design

**Figure 5:**
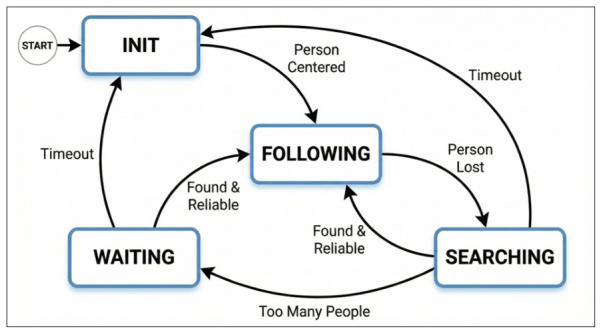
State Machine diagram.

To effectively manage the asynchronous nature of visual detection and ensure safe robot behavior during target loss or ambiguity, the control logic is structured as a Finite State Machine (FSM). This architecture allows the robot to transition deterministically between idle and following behaviors. The different states are shown in Figure 5.

The Finite State Machine (FSM) manages the robot’s behavior through four primary states, as shown in:

- **INIT:** This state handles initialization and target acquisition. The system waits for a human target to enter the field of view. When a target is validated and aligned within the central region ($x_{centre}\pm \tau_{centre}$), the system transitions to FOLLOWING.
- **FOLLOWING:** This is the primary tracking phase. The robot continuously adjusts its velocity to maintain a reference distance ($d_{ref}$) while ensuring that the target remains centered in the camera’s field of view. If tracking is lost, the system immediately shifts to SEARCHING.
- **SEARCHING:** This recovery phase activates immediately upon target loss. The robot executes a rotational search based on the target’s last observed movement direction (left or right). This active scanning is critical for re-acquiring subjects who have moved laterally out of the field of view, a scenario where a stationary wait would likely fail. To prevent indefinite rotation, a timeout mechanism ($\tau_{search}$) resets the system to INIT if the target is not re-acquired within a set time limit.
- **WAITING:** This state manages ambiguity to prevent false locks. If multiple people are detected during a search, the robot pauses all motion. It remains in this state until a single, clear target is identified or the timeout resets the system to INIT.

### 3.3. Visual Perception and Target Tracking

The visual tracking module identifies the target user and estimates their spatial position relative to the robot. The system utilizes the ZED API [42,43] to retrieve raw RGB images, depth maps, and real-time object tracking states. To accommodate the bandwidth limitations of the platform’s USB 2.0 interface, the camera is configured to operate at VGA resolution with a sampling rate of 30 FPS. Experimental validation confirmed that this configuration provides sufficient data quality for real-time processing. Additionally, depth thresholds are calibrated to filter distant background noise, while the neural network is explicitly configured to isolate the PERSON class.

#### 3.3.1. Target Identification

To ensure consistent tracking, the system relies on persistent object identifiers (IDs) that remain constant throughout the duration of tracking. For every frame *k*, the detection module returns a set of detected objects $O_{k}=\left\{o_{1},o_{2},\ldots ,o_{n}\right\}$. Each object $o_{i}$ is characterized by its spatial coordinates, tracking state, detection confidence score $C(o_{i})$, and a unique tracking identifier $ID(o_{i})$.

The tracking logic for the current frame *k* is governed by the following decision stages:

1. **ID Matching:** The algorithm first attempts to locate the specific target object $o_{i}\in O_{k}$ such that $ID\left(o_{i}\right)=ID_{tracked}$, where $ID_{tracked}$ is the identifier of the subject tracked in the previous frame k−1. If the ID matches and the tracking state is valid, the target is confirmed. Conversely, if the previously tracked ID is not found in the current frame ($ID_{tracked}\notin O_{k}$), the system flags the target as lost.
2. **New Target ID Assignment:** A new target ID is assigned if and only if the scene contains exactly one detected person ($|O_{k}|=1$) and the detection confidence $C(o_{i})$ exceeds the minimum reliability threshold $C_{min}$. If it is reassigning in the SEARCHING state, then the threshold is $C_{reassign}$, which is slightly higher for more reliability.
3. **Ambiguity Handling:** If multiple people are detected ($|O_{k}|>1$) while the robot is in the SEARCHING state, the system enters a fail-safe WAITING state. No ID is assigned to prevent the robot from arbitrarily locking onto an incorrect subject. The system waits until the scene resolves to a single, high-confidence candidate.

#### 3.3.2. Depth Estimation Strategy

To accurately estimate the distance to the target, the system employs a Masked Region of Interest (ROI) approach. For the tracked target, the ZED SDK provides a raw depth map and a binary segmentation mask of the identified object M(u,v), where M(u,v)=1 for pixels belonging to the person and 0 otherwise. The raw depth map is preprocessed to ensure numerical stability. Any infinite or undefined (NaN) values detected within the depth data are identified and reset to zero to prevent calculation errors. The target distance $d_{target}$ is computed as the arithmetic mean of the depth map D(u,v) over the valid mask  region:

(1) $$d_{target}=\frac{1}{N}\sum_{u,v}D\left(u,v\right)\cdot M\left(u,v\right)$$

where *N* is the total count of valid mask pixels. Once the target’s position $x_{bb}$ and distance $d_{target}$ are quantified by the perception module, these values serve as the inputs for the velocity controller.

### 3.4. Velocity Control Algorithm

The robot’s motion is governed by simultaneously adjusting its linear and angular velocities based on the user’s relative position in the camera frame. This approach ensures that the robot can react to forward movement and turns at the same time:

- **Linear Control (Distance):** The depth camera continuously estimates the distance to the user. If the user moves away (increasing depth), the robot generates a forward velocity to maintain the reference distance. Conversely, if the distance drops below a safety threshold, the robot actively reverses.
- **Angular Control (Centering):** To handle turns, the system monitors the user’s horizontal alignment. If the user shifts to the left or right of the image center, the robot rotates to realign the optical axis with the target.

While this logic provides the baseline following behavior, simple proportional adjustments were found to be insufficient for elderly care. Consequently, the final algorithm partitions the control logic into specific velocity zones, as discussed in further section, to handle acceleration and stability. The specific tuning parameters and safety thresholds used in the final system are summarized in Table 1.

**Figure 6:**
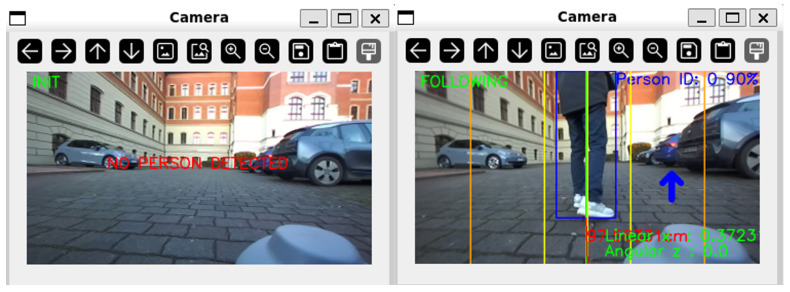
System operation states. (**Left**): The INIT state where the system waits for a valid target. (**Right**): The FOLLOWING state where the target is locked and tracking parameters are active.

Once the person to be tracked is properly positioned at the center of the robot’s view, the system transitions from the INIT state to the FOLLOWING state (Figure 6). In this mode, the robot continuously adjusts the velocity commands $\left(v_{x},\omega_{z}\right)$ to keep the target directly in front and at a consistent and safe distance.

#### 3.4.1. Linear Velocity Control

The linear velocity $v_{x}$ is modulated to minimize the distance error $e_{d}=d_{target}-d_{ref}$, where the ideal following distance $d_{ref}$ is set to 80 cm. The control logic partitions the distance into three distinct behavioral zones:

1. **Stability Zone:** To prevent the robot from oscillating when the user is stationary, the system ignores small deviations $\Delta_{d}$:
  (2) $$v_{x}=0\;\mathrm{if}\;\left|e_{d}\right|<\Delta_{d}$$
  The stability zone ($\Delta_{d}=8$ cm) represents a ±10% tolerance band relative to the 80 cm ideal following distance $d_{ref}$. This deadband is specifically designed to prevent high-frequency control jitter by ignoring minor distance fluctuations. By providing this margin, the system filters out natural human gait oscillations, ensuring that the quadruped does not engage in constant, unnecessary micro-adjustments.
2. **Forward Motion Zone** ($d_{target}>d_{ref}+\Delta_{d}$): The forward behavior is divided into two sub-modes based on how far the user is from the robot.
  - **Catch-up Mode** ($d_{target}>d_{far}$): If the user exceeds a defined far threshold $d_{far}$, the robot engages a high constant velocity $v_{far}$ to close the gap quickly.
    (3) $$v_{x}=v_{far}$$
  - **Proportional Following Mode** ($d_{far}\geq d_{target}>d_{ref}+\Delta_{d}$): In the standard following range, the velocity is proportional to the distance error.
    *Slope Calculation:*
    (4) $$K_{p}=\frac{v_{max}-v_{min}}{d_{far}-d_{ref}}$$
    *Raw Velocity Command:*
    (5) $$v_{raw}=K_{p}\cdot \left(d_{target}-d_{ref}\right)+v_{min}$$
    *Acceleration Ramping:* To prevent sudden jerks, the command is ramped relative to the previous velocity $v_{x}\left(t-1\right)$ using a step size $\delta_{ramp}$:
    (6) $$v_{x}\left(t\right)=\min\left(v_{raw},\;v_{x}\left(t-1\right)+\delta_{ramp}\right)$$
    Calculated velocities are clamped such that $v_{x}\geq v_{min}$.
3. **Backward Motion Zone** ($d_{target}<d_{ref}-\Delta_{d}$): If the user encroaches on the robot’s personal space, the robot reverses. This is handled by a two-stage logic:
  (7) $$v_{x}=\left\{\begin{array}{cc} -v_{crit} & \mathrm{if}\;d_{target}\leq d_{close}\\ -v_{back} & \mathrm{if}\;d_{close}<d_{target}<d_{ref}-\Delta_{d} \end{array}\right.$$
  where $d_{close}$ represents the critical proximity threshold, $v_{crit}$ is the fast retraction speed, and $v_{back}$ is the standard backing speed.

**Figure 7:**
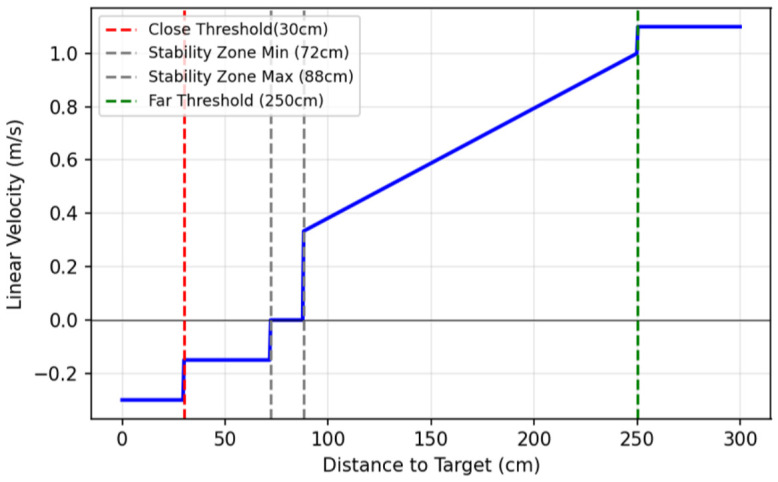
Linear velocity variation plot detailing the tracking bounds and how velocity varies within these bounds. The solid blue line tracks the targeted linear velocity; the dashed red and green vertical lines mark the critical close and far distance limits, respectively; the twin dashed grey vertical lines define the boundaries of the stability zone; and the horizontal solid black line represents the zero-velocity reference baseline.

The complete linear velocity profile, illustrating the transitions between the stability, proportional following, and emergency backing zones, is plotted in Figure 7.

#### 3.4.2. Angular Velocity Control (Centering)

The angular velocity $\omega_{z}$ is controlled to keep the target aligned with the camera’s optical axis. The horizontal error is defined as the deviation between the image center $x_{center}$ and the target’s bounding box center $x_{bb}$:

(8) $$e_{\theta }=x_{center}-x_{bb}$$

The controller employs a debouncing logic to filter out noise, while retaining the ability to react instantly to large deviations.

1. **Debounce Logic with Fast-Track Bypass:** To prevent jitter from minor human swaying, the system utilizes a persistence counter.
  - **Standard Trigger:** If the error $|e_{\theta }|$ exceeds a trigger threshold $\tau_{trigger}$, a counter increments. Correction engages only after $N_{deb}$ consecutive frames.
  - **Fast-Track Bypass:** If the error exceeds a larger safety limit $\tau_{far}$ (target nearing edge of frame), the debounce counter is bypassed, and correction engages immediately.
2. **Dual-Rate Correction:** Once the correction state is active, the robot rotates to minimize the error. The speed is determined by the magnitude of the deviation:
  (9) $$\omega_{z}=\mathrm{sgn}\left(e_{\theta }\right)\cdot \left\{\begin{array}{cc} \omega_{fast} & \mathrm{if}\;|e_{\theta }|>\tau_{far}\\ \omega_{slow} & \mathrm{if}\;\tau_{trigger}<\left|e_{\theta }\right|\leq \tau_{far} \end{array}\right.$$
   where:
  - $\omega_{fast}$ and $\omega_{slow}$ are the configured fast and slow rotational velocities.
  - $\mathrm{sgn}(e_{\theta })$ is set to +1 for an anticlockwise rotation when the target is to the left.
  - $\mathrm{sgn}(e_{\theta })$ is set to -1 for a clockwise rotation when the target is to the right.
3. **Hysteresis Reset:** To prevent oscillation around the trigger threshold $\tau_{trigger}$, the robot continues rotating until the error drops below a distinct reset threshold $\tau_{reset}$ (where $\tau_{reset}<\tau_{trigger}$), at which point the angular deviation and debounce counters are reset.
4. **Searching State**: During SEARCHING state, the robot rotates with a constant angular velocity $\omega_{search}$.

**Figure 8:**
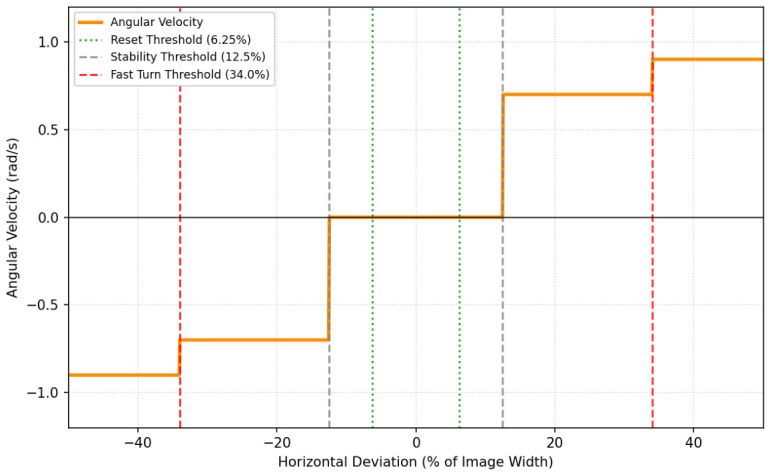
Angular velocity variation plot detailing the velocity variation based on horizontal deviation percentage. The solid orange line indicates the variation of the angular velocity; the twin dashed grey vertical lines outline the initial stability threshold; the twin dotted green vertical lines mark the inner hysteresis reset threshold; the twin dashed red vertical lines highlight the outer fast turn safety thresholds; and the solid horizontal black line provides the zero-rate angular reference axis.

The complete angular velocity profile is plotted in Figure 8.

**Figure 9:**
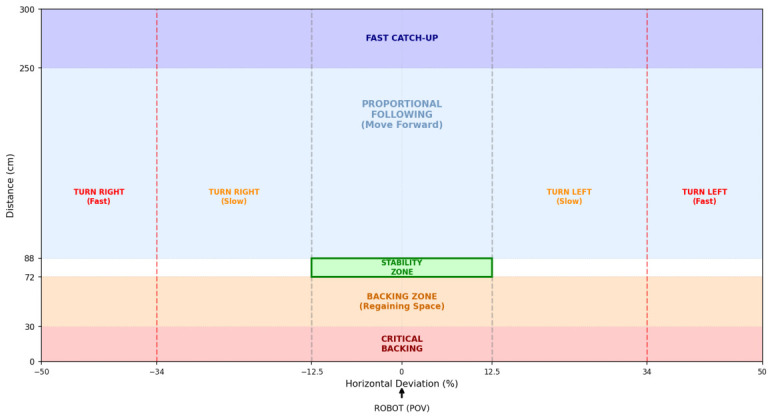
Visual representation of the robot’s field of view (FoV) zoning and internal decision matrix. The distinct color-coded regions define active motion profiles: dark purple (top) indicates the high-speed catch-up zone; light blue marks proportional forward progression; orange denotes the backward motion zone; and light red highlights the critical emergency backing zone. The centrally aligned green bounding rectangle outlines the standstill stability zone. Vertical grey dashed lines mark the horizontal stability thresholds, and vertical red dashed lines distinguish the fast turn transition boundaries. The black arrow beneath the origin clarifies the control system’s perspective relative to the robot’s point-of-view.

This concurrent control strategy results in fluid motion that mimics natural human walking. The resulting behavior is visualized in Figure 9, which defines the robot’s decision matrix. This diagram illustrates how the system seamlessly blends forward and rotational commands to maintain the target within the safety envelope.

### 3.5. Robustness and Safety Mechanisms

Although the velocity controllers ensure smooth motion during ideal tracking, real-world elderly care scenarios involve dynamic clutter and interruptions. Therefore, a supervisory logic layer is required to validate targets before the robot is allowed to move. This section details the specific verification protocols implemented to ensure tracking integrity during target loss and re-acquisition.

#### 3.5.1. Target Re-Acquisition Reliability Protocol

When the robot enters the SEARCHING state, it actively rotates to locate the lost user. During this phase, bystanders may inadvertently enter the frame. To prevent the robot from locking onto an incorrect subject, the system enforces a strict ”Reliability Check” before restoring the FOLLOWING state. A candidate subject must satisfy two consistency constraints simultaneously:

1. **Depth Consistency Constraint:** The system maintains a short-term memory of the user’s last known depth $d_{last}$. Upon detecting a new candidate $O_{new}$ with depth $d_{new}$, the system calculates the displacement:
  (10) $$\Delta_{depth}=\left|d_{new}-d_{last}\right|$$
  The candidate is rejected if $\Delta_{depth}>\tau_{depth}$. This filter assumes that a target cannot physically displace more than $\tau_{depth}$ meters during the short interval between target loss and re-acquisition. The depth consistency threshold ($\tau_{depth}=70$ cm) is derived from the maximum expected human displacement during a short search interval (≈1 s). This selection is supported by recent geriatric gait studies of community-dwelling older adults with mobility limitations, which report a mean walking speed of 0.77 m/s [44]. Assuming this representative speed, the maximum physical displacement of a target is approximately 0.77–0.8 m per second. By setting $\tau_{depth}$ to 70 cm, the system enforces a strict kinematic bound that filters out bystanders appearing at different depth planes while remaining sufficiently flexible to re-acquire the primary user even after a momentary loss of tracking.
2. **Geometric Entry Constraint:** This geometric filter exploits the correlation between the robot’s search direction and the expected location of the target’s re-entry into the Field of View (FoV). If the robot is rotating to find a target, the valid user is physically required to appear from the leading edge of the turn:
  - **Rightward Search:** If the robot is rotating Right (searching for a target lost to the right), the candidate is considered valid only if they appear in the rightmost part of the image width, defined by $\tau_{entry}$.
  - **Leftward Search:** Conversely, if rotating Left, the candidate must appear in the leftmost part of the image width, defined by $\tau_{entry}$.
  Any detection appearing in the ”Invalid Zone” (the opposite side or center) during a search is classified as a bystander and ignored.

#### 3.5.2. Ambiguity Failsafe

When the perception module flags an ambiguous frame ($|O_{k}|>1$) during a search, the robot enters the WAITING state. Velocities are immediately clamped to zero ($v_{x}=0,\omega_{z}=0$) to prevent incorrect targeting.

## 4. Experimental Results

### 4.1. Experimental Setup

The framework was developed and refined through an iterative prototyping process, including multiple functional pilot tests in both outdoor settings and care-facility workshops. While these general tests confirmed the overall reliability of the system, the formal evaluation reported in this study was conducted in a controlled outdoor environment to isolate core control behaviors from indoor environmental clutter. Consequently, this study validates the fundamental path-following logic; active obstacle avoidance is outside the current scope and is reserved for future integration with the ROS Navigation stack [45].

While the target demographic for this framework is the elderly, the initial validation was performed with a healthy adult subject in a controlled outdoor environment as a mandatory safety and ethical prerequisite. The robot’s onboard ZED 2 camera captured depth and RGB data, which was processed by an external laptop running the ROS control node.

**Configuration and Sensing:** To prioritize real-time system responsiveness and maximize the control loop frequency, the ZED depth engine was configured in ’PERFORMANCE’ mode. While the system was also validated using the computationally intensive ’ULTRA’ mode during preliminary testing, the ’PERFORMANCE’ setting provided sufficient depth precision for robust person detection while ensuring a lower computational overhead for the reported field trials.

**Figure 10:**
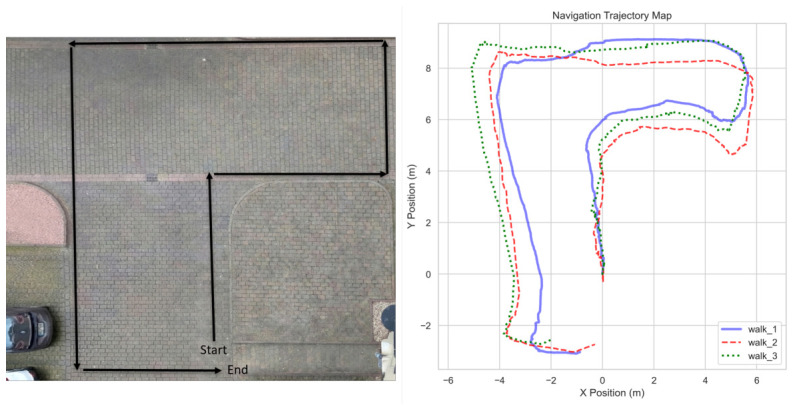
Experimental validation. (**Left**): Aerial view of the testing ground showing the rectangular path plan (start to end). (**Right**): The recorded odometry trajectory map for three separate trials (walk_1 to walk_3), demonstrating consistent navigation through 90-degree turns.

**Evaluation Methodology:** The experimental evaluation was divided into two distinct phases:

- **Inter-trial Variability and Repeatability:** A parking lot was selected to provide a rectangular path with several 90° turns to evaluate tracking stability during rotation. Three representative trials (N=3) were logged to analyze the repeatability of the path-following and distance-maintenance logic. The specific rectangular path used for the repeatability trials is illustrated in Figure 10.
- **System Characterization Benchmarks:** Separate specialized tests were conducted to evaluate core system benchmarks, including processing latency (comparing headless vs. graphical modes) and the controller’s step response to sudden target movements.

### 4.2. Inter-Trial Variability and Repeatability

The system underwent field testing to validate core control behaviors by logging command velocities, visual odometry, and system states in real time. To evaluate the deterministic nature of the framework, inter-trial variability was analyzed across three high-fidelity logs. As summarized in Table 2, the results demonstrate a remarkably low standard deviation in system latency (±0.07 ms), confirming a highly stable processing loop. While the mean distance error varied between trials (41.21 cm to 51.96 cm), this is attributed to varying initial human starting positions (ranging from 55 cm to 123 cm) rather than algorithmic drift.

The spatial consistency of these results can be observed in Figure 10, which provides a visual map of the recorded odometry trajectories across all three trials. Despite varying start points, the system consistently converged to the target following distance and demonstrated robust recovery during a momentary tracking loss in Walk 1, which was autonomously resolved in 0.607 s.

**Figure 11:**
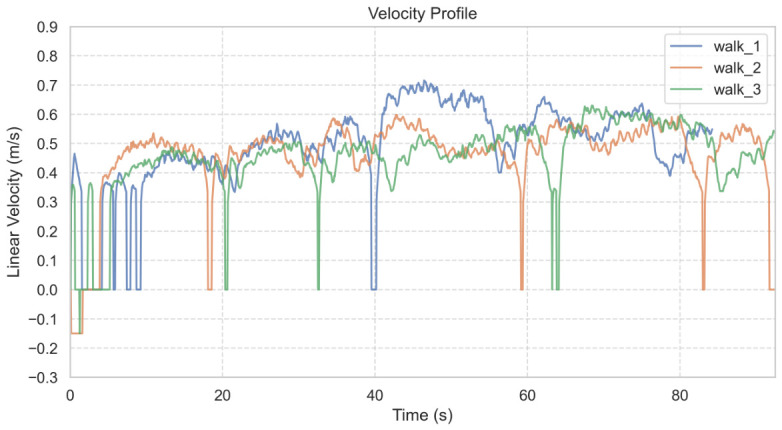
Linear velocity profile showing smooth acceleration and the absence of high-frequency jitter.

The robot’s velocity consistency across these trials is further illustrated in the linear velocity profile (Figure 11). To quantify tracking performance, average velocities across different path segments were analyzed (Table 3). The human walking speed was derived from the time taken to traverse known path segment lengths. The results indicate a high degree of synchronization, particularly in active segments (Paths 2–5), where the robot maintained an average velocity between 0.47 and 0.54 m/s, closely matching the human target (0.51–0.55 m/s).

The lower velocity observed in Path 1 (0.37 m/s) is attributed to the system’s initialization phase and startup inertia. However, this data point also empirically demonstrates the controller’s stability at lower speeds. Mathematically, the proportional control logic defined in Equation (5) ensures that linear velocity scales downward as the distance error $e_{d}$ decreases. This allows the system to remain synchronized with lower gait speeds by adjusting the pace dynamically rather than exhibiting jerky ’start-stop’ behavior.

### 4.3. System Characterization Benchmarks

While the previous section established the spatial repeatability of the framework, this section evaluates the underlying technical benchmarks that ensure deterministic operation. The evaluation focused on four key performance metrics: system latency, control stability, safety enforcement, and search autonomy. These characterization tests were conducted to quantify the efficiency of the “Headless” architectural shift and the responsiveness of the velocity controller to dynamic target inputs.

#### 4.3.1. System Latency and Processing

**Figure 12:**
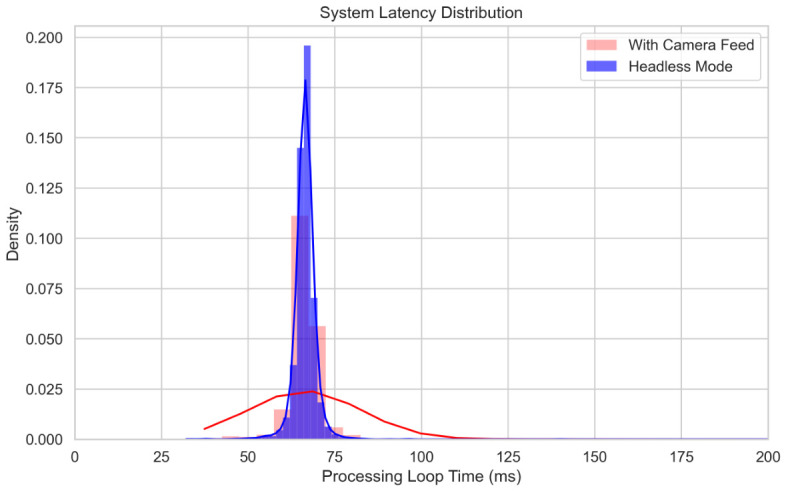
System latency distribution comparing Headless and active Camera Feed operating modes. The blue shaded histogram bars and tightly bound solid blue curve display the processing loop time frequencies and kernel density estimation (KDE) profile for the optimized Headless mode; the red shaded bars and broad solid red curve represent the frequency distribution and corresponding KDE profile when the graphical camera feed is active.

To prioritize real-time responsiveness, the system was operated exclusively in Headless mode for all navigation and stability trials to maximize control frequency. As illustrated in the latency distribution (Figure 12), transitioning to a Headless architecture significantly improved system performance.

The mean processing loop time was reduced to 66.5±4.3 ms, compared to 72.0±62.1 ms when the graphical camera feed was active. More critically, the Headless mode effectively eliminated massive latency spikes, with observed maximum freezes exceeding 1.5 s, which occurred during graphical rendering. By removing these computational bottlenecks, the framework achieved a 93.08% reduction in timing jitter (based on the reduction of standard deviation from 62.1 ms to 4.3 ms).

#### 4.3.2. Distance Maintenance and Stability

The integration of the acceleration ramping function effectively eliminated the abrupt “start-stop” jitter characteristics observed in the native platform. During sudden stops, the specific “Stability Zone” logic prevented unnecessary micro-adjustments.

**Figure 13:**
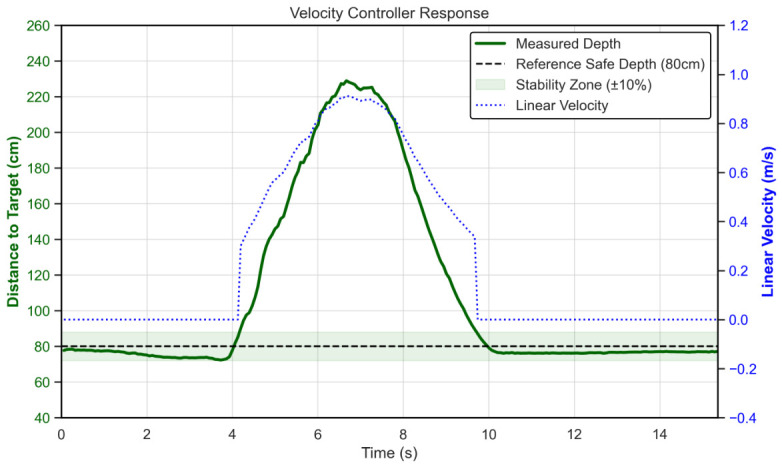
Controller step response. The robot smoothly decelerates and settles at the target distance (80 cm) when the user stops.

**Figure 14:**
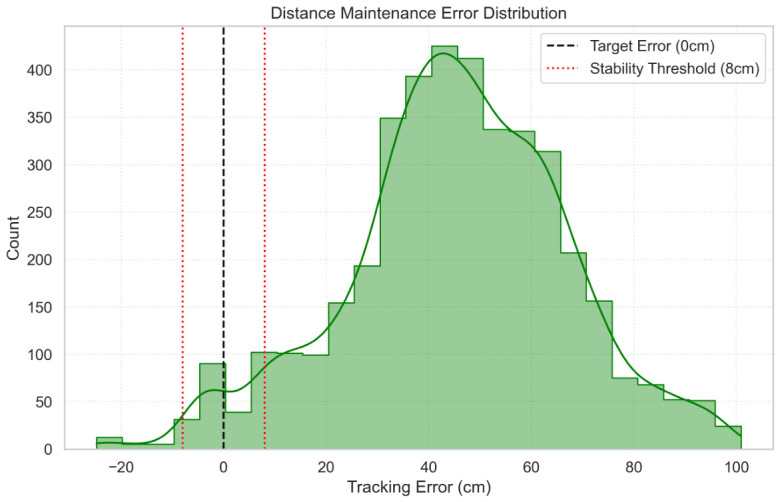
Distribution of distance maintenance error. The shaded green bars display the raw error frequency histogram, while the solid green curve denotes the corresponding continuous kernel density estimation profile; the vertical dashed black line represents the ideal zero tracking error target, and the twin dotted red lines frame the deadband stability threshold boundaries. The skew towards positive error indicates safe lagging rather than unsafe crowding.

Figure 13 illustrates the response when the user made a few steps from a standstill; the robot settles into the stable zone, with a minimum recorded depth of 72.3 cm, within a settling time of approximately 9.67 s, successfully avoiding collision or oscillation. The error distribution (Figure 14) confirms that the robot predominantly maintains the target distance within the safe tracking envelope.

#### 4.3.3. Tracking Robustness and Safety

**Figure 15:**
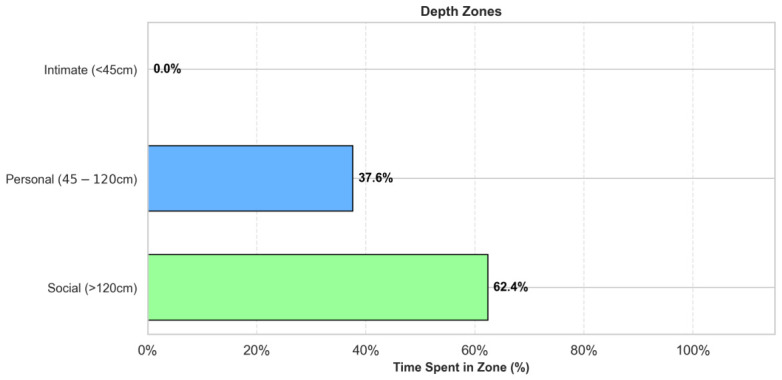
Proxemics safety analysis. The robot successfully avoided the ’Intimate’ collision zone (0.0% time) throughout the trials.

Safety was evaluated using Hall’s proxemics zones. Figure 15 confirms that the robot spent 0.0% of the experiment time in the “Intimate Zone” (<45 cm), strictly adhering to the “Personal” and “Social” safety distances.

**Figure 16:**
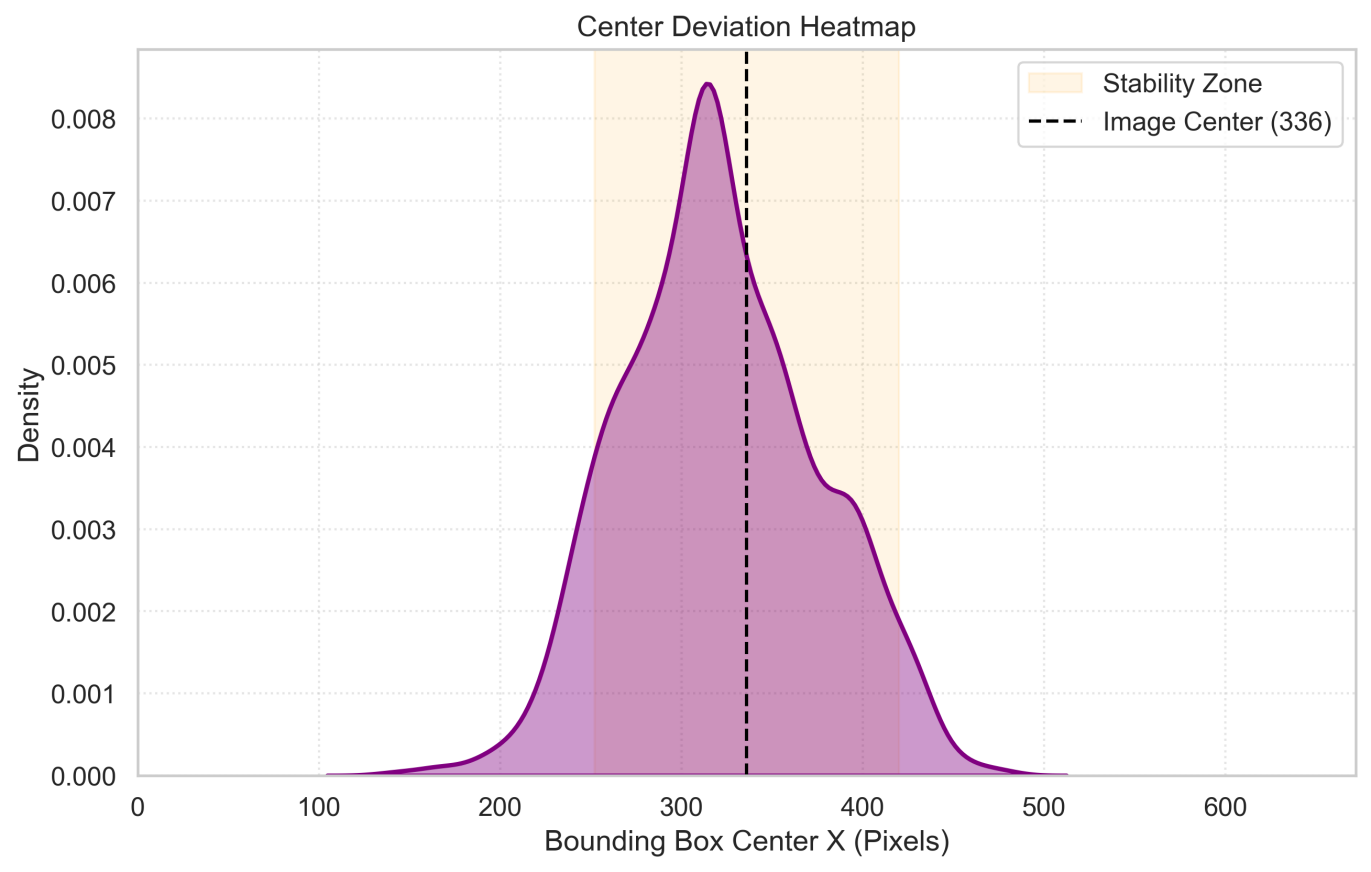
Heatmap of target centroid deviation relative to the frame midline. The purple curve and shaded area display the continuous kernel density estimation profile of the deviation; the vertical dashed black line marks the image center; and the shaded vertical background block highlights the deadband stability zone. The high density at the center (x=336) validates the angular tracking loop.

The centering logic also performed as expected. The heatmap in Figure 16 shows that the target was consistently maintained near the image center (x=336), validating the angular tracking loop.

**Figure 17:**
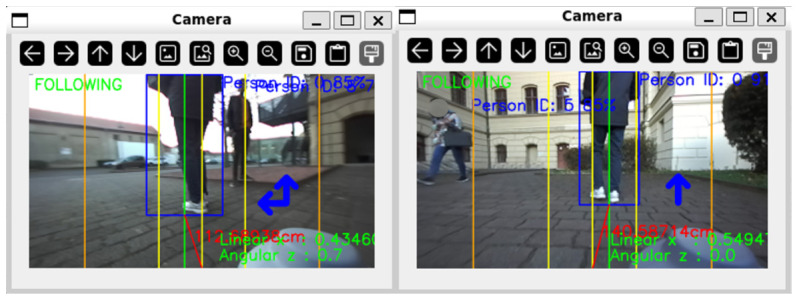
Robustness validation: The system maintains a lock on the original Target (ID 0) while ignoring a Bystander (ID 5) crossing the field of view.

Furthermore, the reliability verification logic was qualitatively validated. As shown in Figure 17, when a bystander (ID: 5) crossed the robot’s path, the system rejected the false target by strictly enforcing the depth consistency check, maintaining lock on the original target (ID: 0).

#### 4.3.4. Search and Recovery

**Figure 18:**
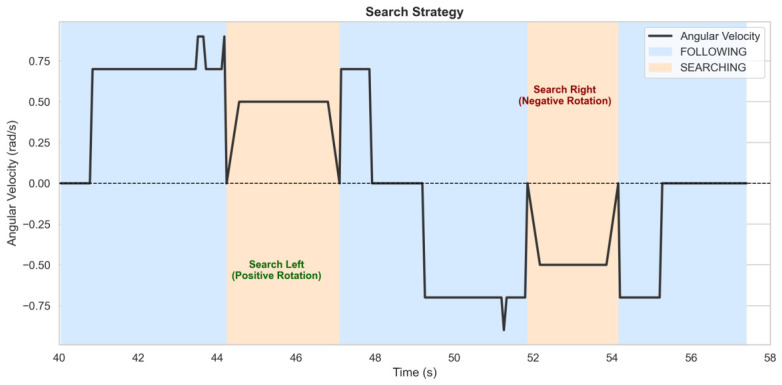
Autonomous search strategy. The plot shows the angular velocity reacting to target loss by initiating a bi-directional search.

The directional memory feature was critical for navigation. Referring back to Figure 10 (Right), the trajectory map highlights the successful negotiation of 90-degree turns without losing the target. Figure 18 details the specific search logic: upon target loss, the robot automatically initiated a rotational search in the correct direction (Left/Right) based on the last known motion vector, allowing for successful re-acquisition.

### 4.4. Discussion

The experimental results demonstrate that the proposed custom architecture successfully addresses the specific limitations of the native firmware identified in Section 1. By shifting the design focus from high-dynamic sports to safe interaction and reliable following, several key improvements were realized. To quantify these advancements, Table 4 provides a comparative summary of the performance characteristics observed during preliminary testing of the native system versus the results of the proposed framework.

The comparison with the native Go1 follow mode is based on qualitative observations from preliminary testing. Due to the closed-source, “black-box” nature of the native system, a controlled quantitative benchmark was not feasible. Initial tests indicated unreliable behaviour, so research did not proceed with a numerical comparison. Instead, the poor baseline performance of the proprietary firmware motivated the independent development and rigorous validation of the proposed framework. The improvements summarized in the Table 4 are further detailed as follows:

- **Stability over Agility:** While the native system prioritizes rapid movement, the proposed velocity control strategy eliminates the “start-stop” jitter observed during preliminary testing. The ramping function and stability zones ensure the fluid motion required for pacing elderly users.
- **Deterministic Behavior:** Unlike the proprietary “black-box” nature of the native system, the Finite State Machine (Figure 5) provides transparent and predictable decision-making. Features such as the bi-directional search ensure that the robot recovers from target loss in a logical manner, rather than failing opaquely.
- **Explicit Safety Verification:** The native system lacks configurable safety margins. The proposed system explicitly enforces Hall’s proxemics, verifying 0.0% intrusion into the Intimate Zone (<45 cm) and utilizing depth consistency checks to reject bystanders (Figure 17), a critical capability for deployment in shared care facilities.

As a mandatory safety and ethical prerequisite, this study utilized a healthy adult subject to establish a baseline evaluation of control behavior under controlled conditions before deployment in sensitive populations.

## 5. Conclusions

This paper presented a robust, vision-based, person-following framework for the Unitree Go1, specifically tailored to prioritize the motion smoothness and predictability required for elderly care. By bypassing the native firmware in favor of a custom ROS-based architecture and ZED 2 perception, the system successfully addresses critical stability and latency limitations. Functional validation confirmed that the proposed state machine and velocity zoning strategies ensure deterministic target acquisition, smooth acceleration, and reliable recovery logic. These findings establish a stable technical foundation for the future development of robotic walking partners.

## 6. Limitations and Future Work

The proposed framework represents a foundational prototype within the EduXBot project, focusing on deterministic, vision-based, person-following behavior. The system was evaluated in a controlled outdoor environment to isolate core control performance. As such, the results do not fully reflect real deployment conditions in elderly care settings, such as confined indoor spaces or cluttered environments. It is important to note that the experimental validation was conducted with a healthy adult subject, and no trials with elderly users were performed. Additionally, the number of evaluation trials is limited, and the reported results should be interpreted as indicative of the technical baseline rather than statistically conclusive for all populations.

Furthermore, the current iteration of the system does not yet include integrated obstacle avoidance, advanced navigation capabilities, or multi-person tracking. The long-term robustness of the system, particularly under extended headless operation, also remains to be evaluated. Despite these limitations, the prototype was demonstrated on multiple occasions where consistent and stable following behavior was observed, and informal user feedback indicated positive acceptance of the system’s functionality and predictability. This aligns with prior studies suggesting that assistive robots are perceived positively when they enhance user independence and provide reliable support in mobility-related tasks.

While the current phase of the project has established a verified technical baseline, the transition of the platform from a functional prototype to a viable assistive tool for geriatric care requires further development. Future work is intended to address existing limitations by integrating the framework with navigation stacks for active collision avoidance and extending validation to more complex, unstructured indoor environments. Ultimately, the proposed next steps involve conducting formal user studies with elderly participants in clinical contexts to assess usability, safety, and psychological acceptance. These subsequent stages are essential for refining the system’s interactive capabilities before broader deployment.

## Figures and Tables

**Table 1:** Configured Control Parameters for Velocity, Stability, and Thresholds.

Symbol	Value	Description
$d_{ref}$	80 cm	Target following distance
$\Delta_{d}$	8 cm	Stability threshold
$d_{close}$	30 cm	Emergency backing threshold
$d_{far}$	250 cm	Max distance threshold
$x_{center}$	336 px	Image center pixel
$\tau_{trigger}$	12.5%	Angular error threshold
$\tau_{far}$	34%	Fast rotation threshold
$\tau_{centre}$	12.5%	Initial centre threshold
$\tau_{reset}$	6.25%	Angular error reset threshold
$\tau_{depth}$	70 cm	Re-acquisition depth limit
$\tau_{entry}$	30%	Valid search entry width
$\tau_{search}$	3 s	Search state timeout threshold
$N_{deb}$	2	Angular deviation debounce threshold
$v_{far}$	1.1 m/s	Too far forward velocity
$v_{max}$	1.0 m/s	Max forward velocity
$v_{min}$	0.3 m/s	Min forward velocity
$v_{back}$	0.15 m/s	Standard backing speed
$v_{crit}$	0.3 m/s	Fast retraction speed
$\delta_{ramp}$	0.025 m/s	Velocity ramp step
$\omega_{slow}$	0.7 rad/s	Minor correction speed
$\omega_{fast}$	0.9 rad/s	Major correction speed
$\omega_{search}$	0.5 rad/s	Search state speed
$C_{min}$	55%	Minimum confidence for detection
$C_{reassign}$	60%	Minimum confidence for reassign

**Table 2:** Statistical Summary of Inter-trial Variability and Performance.

Metric	Walk 1	Walk 2	Walk 3	Aggregate (μ±σ)
System Latency (ms)	66.55	66.43	66.42	66.47 ± 0.07
Mean Distance Error (cm)	51.96	43.87	41.21	45.68 ± 5.60
Mean Center Error (px)	−22.96	−12.43	−10.77	−15.39 ± 6.61
Tracking Losses (qty)	1	0	0	1 (Total)
Recovery Time (s)	0.607	-	-	0.607 (Max)

**Table 3:** Comparison of Human vs. Robot Average Velocities across Path Segments (N = 3 Trials).

Path Segment	Avg Robot Vel (m/s)	Avg Human Vel (m/s)
1	0.37	0.54
2	0.47	0.51
3	0.48	0.40
4	0.53	0.55
5	0.54	0.51
6	0.48	0.22

**Table 4:** Architectural and Performance Comparison: Native Go1 Firmware vs. Proposed Framework.

Feature	Native Follow Mode	Proposed Framework
Motion Stability	Observed instability; robot continues moving when the target is stationary.	Deterministic stability zone; robot settles at 80 cm without oscillation.
Sensing Logic	Active method requiring a wearable tag; does not detect surrounding bystanders.	Passive vision-based method; explicitly detects and verifies target via depth.
System Setup	Closed-source “black box”; high setup complexity and unclear mode selection.	Open-source ROS architecture; transparent control loop of 66.5±4.3 ms.
Target Recovery	Based solely on tag signal orientation; lacks visual spatial context.	FSM-based search using directional memory and bystander rejection.
Obstacle Avoidance	Built-in system; inconsistent behavior during follow mode in preliminary tests.	Current focus on path-following; integration with ROS NavStack is planned.

## Data Availability

The original contributions presented in this study are included in the article. Further inquiries can be directed to the corresponding author.

## Abbreviations

The following abbreviations are used in this manuscript:

WHO	World Health Organization
SARs	Socially Assistive Robots
EduXBot	Educational Exploration Robot Application Platform
UDP	User Datagram Protocol
Go1	Unitree Go1 quadruped robot
ROS	Robot Operating System
SDK	Software Development Kit
LIDAR	Light Detection and Ranging
SLAM	Simultaneous Localization and Mapping
API	Application Programming Interface
UWB	Ultra-Wideband
RFID	Radio Frequency Identification
YOLO	You Only Look Once
RGB	Red, Green, Blue
RGB-D	Red, Green, Blue plus Depth
FPS	Frames Per Second
USB	Universal Serial Bus
VGA	Video Graphics Array
FoV	Field of View
ID	Identifier
FSM	Finite State Machine
ROI	Region of Interest
NaN	Not a Number
